# Emptying of Intracellular Calcium Pool and Oxidative Stress Imbalance Are Associated with the Glyphosate-Induced Proliferation in Human Skin Keratinocytes HaCaT Cells

**DOI:** 10.1155/2013/825180

**Published:** 2013-08-29

**Authors:** Jasmine George, Yogeshwer Shukla

**Affiliations:** Proteomics Laboratory, Indian Institute of Toxicology Research (CSIR), Mahatma Gandhi Marg, Lucknow, Uttar Pradesh 226001, India

## Abstract

We demonstrated that glyphosate possesses tumor promoting potential in mouse skin carcinogenesis and SOD 1, calcyclin (S100A6), and calgranulin B (S100A9) have been associated with this potential, although the mechanism is unclear. We aimed to clarify whether imbalance in between [Ca^2+^]_*i*_ levels and oxidative stress is associated with glyphosate-induced proliferation in human keratinocytes HaCaT cells. The [Ca^2+^]_*i*_ levels, ROS generation, and expressions of G1/S cyclins, IP_3_R1, S100A6, S100A9, and SOD 1, and apoptosis-related proteins were investigated upon glyphosate exposure in HaCaT cells. Glyphosate (0.1 mM) significantly induced proliferation, decreases [Ca^2+^]_*i*_, and increases ROS generation in HaCaT cells, whereas antioxidant N-acetyl-L-cysteine (NAC) pretreatment reverts these effects which directly indicated that glyphosate induced cell proliferation by lowering [Ca^2+^]_*i*_ levels via ROS generation. Glyphosate also enhanced the expression of G1/S cyclins associated with a sharp decrease in G0/G1 and a corresponding increase in S-phases. Additionally, glyphosate also triggers S100A6/S100A9 expression and decreases IP_3_R1 and SOD 1 expressions in HaCaT cells. Notably, Ca^2+^ suppression also prevented apoptotic related events including Bax/Bcl-2 ratio and caspases activation. This study highlights that glyphosate promotes proliferation in HaCaT cells probably by disrupting the balance in between [Ca^2+^]_*i*_ levels and oxidative stress which in turn facilitated the downregulation of mitochondrial apoptotic signaling pathways.

## 1. Introduction

Glyphosate, “an organophosphate herbicide,” is the active component of Roundup and considered being innocuous whether alone or in combination with its formulation products such as surfactants under regular usage or chronic exposure in earlier testing approach in humans [1, 2]. However, lately acute toxic activity of glyphosate at lethal concentration has been demonstrated in fish or other aquatic organisms [3, 4]. Some case-control studies suggested an association between glyphosate use and the risk of non-Hodgkin lymphoma among men [5].

The cellular reply to carcinogens/toxicants is intricate, and considerable effort is put into defining the network of proceedings going on in the cell to preserve genomic stability and avert carcinogenesis. Intracellular Ca^2+^ signaling is vital in the regulation of multiple cellular processes, including development, proliferation, secretion, gene activation, and cell death [6–8]. The development of these Ca^2+^ signals is reliant on many cellular Ca^2+^-binding and Ca^2+^-transporting proteins, existing in the several cell compartments of which the endoplasmic reticulum (ER) forms the main intracellular Ca^2+^ store [9]. The S100 family is Ca^2+^-binding proteins comprises around 20 genes that are positioned in a cluster on chromosome 1q21 [10]. Expression of several S100 proteins appears to be transformed in different types of cancers [11]. Particularly, calcyclin (S100A6) and calgranulin B (S100A9) containing 2 EF-hand Ca^2+^-binding motifs are presently enticing ample attention for their extensive variety of potential intracellular along with extracellular functions [12]. For instance, both proteins have been proposed to be involved in the regulation of cell proliferation, apoptosis, and motility through Ca^2+^-dependent signaling pathways [13]. Likewise, both of them have been defined to play a part in the pathogenesis of epidermal disease involving melanoma or epithelial skin cancer and inflammation [14]. The expression pattern of S100A6 and S100A9 expressions was found to be considerably enhanced in some tumor tissues like hepatocellular carcinoma [15], lung cancer [16], colorectal cancer [17], and melanoma [18].

Modification in the ionized intracellular Ca^2+^ concentration has been associated with the production of reactive oxygen species (ROS) [19]. ROS (O_2_^−^, hydrogen peroxide (H_2_O_2_), ROOH, HO, etc.) are commonly considered as toxicants that persuade numerous toxic effects, like cell dysfunction, death, or malignant transformation. Oxidative stress is the consequence of the disparity between ROS generation and the cellular antioxidant capacity. It is well-documented that substantial oxidative stress brings out severe harm to lipids, proteins, sugars, and nucleic acid bases, which compromises cell viability and functions [20–22]. In experimental models by means of cell lines, it was displayed that ROS generation and consequent oxidative stress add to cancer progress through arrange of interconnected signals [23]. To bestow, shield against the oxidative stress, the skin is equipped with quite a few enzymatic antioxidants, like superoxide dismutase (SOD), catalase, and many peroxidases [24]. SOD is a tumor suppressor protein that upsurges the dismutation rate of superoxide anion (O_2_^−^) to H_2_O_2_ by 3 to 4 orders of magnitude over unstructured dismutation and prevents cancer cell growth *in vitro* [25, 26]. CuZnSOD (SOD 1) is an Mr 32,000 dimeric SOD family protein that is confined in the cytoplasm [27]. Reports suggest that SOD 1 might be involved in cancer cell invasion and metastasis [28, 29].

Previously, we have reported that glyphosate potentially causes tumor promotion in mouse skin carcinogenesis and S100A6, S100A9, and SOD 1 are associated with this tumor promotion [30]. However, the mechanism behind glyphosate-induced tumor promotion is not fully understood. This prompted us to explore the possibility whether deregulation of Ca^2+^ homeostasis and oxidative stress are playing a role in the proliferation activity of glyphosate. For this, we have studied the underlying mechanism using human skin keratinocyte, HaCaT, cells as an *in vitro* model. These cells were derived from a spontaneously immortalized human keratinocyte. They are a nontumorigenic epidermal cell line that exhibits many of the morphological and functional properties of normal human keratinocytes. The use of HaCaT cells has the good advantage of providing an almost unlimited supply of identical cells, assuring high reproducibility; this cellular model also possesses the enzymatic equipment to bioactivated or detoxifies xenobiotics [31].

## 2. Materials and Methods

### 2.1. Chemicals

The commercial formulation of the herbicide glyphosate (N-phosphonomethyl-glycine) Roundup Original (glyphosate 41%, polyethoxethyleneamine (POEA) *≅*15%—Monsanto Company, St. Louis, MO, USA) was used. It contains glyphosate 360 g/L (acid equivalent) present as the isopropylamine salt and was procured from local market. 2′,7′-Dichlorodihydrofluorescein diacetate dye (H_2_DCF-DA), propidium iodide (PI), 3[4-Dimethylthiazol-2-yl]-2-5-diphenyl tetrazolium bromide (MTT), 4,6-diamidino-2-phenylindole (DAPI), and N-acetyl-L-cysteine (NAC) were purchased from Sigma Chemical Company (St. Louis, CA, USA). Cyclin D1, cdk 4, cdk 6, cyclin E, cdk 2, caspase 3, caspase 9, cytochrome c, apoptotic protease-activating factor-1 (Apaf-1), and *β*-actin antibodies were procured from Cell Signaling Technology (Beverly, MA, USA). Bax, Bcl-2, PCNA (proliferating cell nuclear antigen), BrdU (5-bromo-2′-deoxyuridine), 1 inositol-1,4,5-trisphosphate receptor type 1 (IP_3_R1), S100A6, S100A9, and SOD1 antibodies were procured from Santa Cruz Biotechnology Inc., Europe. Fluorescein isothiocyanate (FITC), rhodamine, and horseradish peroxidase (HRP) conjugated anti-mouse/anti-rabbit secondary antibodies were procured from Bangalore Genei (Bangalore, India) and Cell Signaling Technology, Inc. (Danvers, MA, USA), respectively. The polyvinylidene fluoride (PVDF) membrane was obtained from Millipore (Bedford, MA, USA). All other chemicals were of analytical grade of purity and were procured locally.

### 2.2. Cell Culture

HaCaT cells were procured from National Centre for Cell Science, Pune, India, and cultured in calcium free-Dulbecco's modified Eagle's medium (DMEM) (catalog number 21068-028, Invitrogen, Carlsbad, CA, USA) supplemented with 10% chelex-treated fetal bovine serum (FBS), 1% penicillin streptomycin (Gibco Lifetech, Karlsruhe, Germany). Cells were maintained in a humidified atmosphere of 95% air and 5% CO_2_ at 37 degrees in incubator. 

### 2.3. Cell Proliferation Assay by MTT

To evaluate the dose-response effect of glyphosate on the growth and proliferation of HaCaT cells, the MTT assay was performed as described earlier [32]. Briefly, 1 × 10^4^ cells per well were plated in 96-well flat-bottomed microplate and treated with glyphosate (0.01, 0.025, 0.05, 0.1, 0.25, 0.5, and 1 mM) for 24, 48, and 72 h. To address the antiproliferative potential of NAC, HaCaT cells were alone and preincubated with NAC (10 and 20 mM) followed by a 72 h incubation with glyphosate (0.1 mM). After the incubation, the cells were washed twice with PBS, and 200 *μ*L of culture medium containing 5 mg/mL of MTT dye was added to each well and incubated further for 4 h. The medium containing MTT dye was then replaced with 200 *μ*L of dimethyl sulfoxide (DMSO). The plates were then agitated for 10 min, and the optical density (OD) was measured at 540 nm using microplate reader (FLUOstar Omega-BMG Labtech). 12-O-tetradecanoyl-phorbol-13-acetate (TPA) (10 nM) was used as a positive control group. 

### 2.4. Immunofluorescence Studies

HaCaT cells proliferation was examined using immunofluorescence staining for PCNA and BrdU. Briefly, HaCaT cells were incubated with glyphosate (0.01, 0.1 mM) and TPA (10 nM) for 72 h. Then, BrdU (10 mM) was added to the medium, and the cells were incubated for 4 h. This exposure was followed by fixing with 4% paraformaldehyde in PBS for 7 min, permeabilization with 0.1% Triton-X-100 in PBS for 7 min, and blocking with 3% BSA in PBS for 1 h with gentle agitation. Further, the cells were incubated with BrdU and PCNA primary antibody for overnight and secondary rhodamine antibody for BrdU and FITC antibody for PCNA at room temperature for 1 h each. The cells were then washed in PBS and counterstained with DAPI. Fluorescence microscopy was performed using olympus IX51 (Olympus America Inc., Center Valley, PA, USA), and images were acquired with the help of software Image-Pro Express. 

### 2.5. Treatment of Cells

Glyphosate stock solution (1 mM in the medium) was prepared in the medium and diluted further in fresh medium to achieve desired final concentration for treatment of cells. Cells that served as vehicle controls were incubated with the medium only. The cells were harvested by trypsinization, washed twice with cold PBS to remove residual medium, and processed as per the requirement of the following assays.

### 2.6. Intracellular Ca^2+^ Measurement

Intracellular Ca^2+^ concentration was measured with the Ca^2+^ indicator dye Fura-2 AM (Sigma Aldrich, USA). Cells were seeded at a density of 16000 cells/well in completed growth medium in a 96-well plate. Next day, they were loaded with Fura-2 AM in loading solution (125 mM NaCl, 5 mM KCl, 1.2 mM MgSO_4_, 1.2 mM KH_2_PO_4_, 2 mM CaCl_2_, 6 mM Glucose, and 25 mM Hepes (pH-7.0)) at 37°C under CO_2_ incubator for 30 min. Excess dye was removed by rinsing twice with wash buffer (NaCl, KCl, NaHCO_3_, and Glucose). Thereafter, cells were treated with NAC (20 mM), glyphosate (0.1 mM), and TPA (10 nM) for 6 h, respectively, harvested, and washed twice with wash buffer. For NAC treatment, cells were preincubated with 20 mM NAC before treating with the same concentration of glyphosate and TPA. Fluorescence was monitored every 10 min at the excitation wavelength of 355 and 380 nm and the emission wavelength of 510 nm at each time point (0–6 h) by microplate reader (FLUOstar Omega-BMG Labtech). All results showed are representative experiments from three separate experiments under the same conditions and by a same procedure at each time point. To convert fluorescent values into absolute Ca^2+^ concentration, calibration was performed at the end of each experiment. The concentration of intracellular Ca^2+^ concentration was calculated using the following formula:

(1) $$\begin{array}{c} {\left[\text{C}{\text{a}}^{2+}\right]}_{i}=\frac{\text{Kd}X\left(F_{\text{o}}-F_{\min}\right)}{\left(F_{\max}-F_{\text{o}}\right)}, \end{array}$$

where Kd is the dissociation constant of the Ca^2+^-bound Fura-2 AM complex (224 nM/L), and *F*_o_ is excitation obtained at the ratio generated by 355/380. *F*_max⁡_ corresponds to the maximum fluorescence obtained by treating cells with 0.1% Triton X-100, and *F*_min⁡_ represents the minimum fluorescence of the cells treated with 5 mM EGTA.

### 2.7. Measurement of ROS Generation

ROS generation was monitored by using H_2_DCFH-DA as described by earlier [33] with slight modifications. Briefly, cells were seeded at a density of 1 × 10^4^ cells/well in a 96-well plate, respectively. The next day, cells were treated with N-acetyl-cysteine (NAC) (20 mM), glyphosate (0.1 mM), TPA (10 nM), and H_2_O_2_ (100 mM) for 24 h followed by incubation with 10 *μ*M H_2_DCFH-DA at 37°C for 30 min in dark. For NAC treatment, cells were preincubated with 20 mM NAC for 1 h after synchronization before treating with the same concentration of glyphosate, TPA, and H_2_O_2_. Fluorescence was measured through a spectrofluorometer by using 507 nm as excitation and 530 nm as emission wavelengths. For each experiment, fluorometric measurements were performed in triplicate, and data is presented as the percentage of vehicle control cells.

### 2.8. Cell-Cycle Analysis

For cell-cycle analysis, the cells were prepared as described earlier [34]. Briefly, vehicle, positive control, and glyphosate (0.1 mM) exposed cells (for 24 h) were washed with PBS and centrifuged at 200 ×g for 10 min at 4°C. The pellet was fixed in 1 mL of  70% ice-cold ethanol for 30 min and resuspended in 50 *μ*g/mL PI with RNase A (100 *μ*g/mL) followed by incubation for 30 min in dark. The samples were acquired and analyzed on flow cytometer using “CellQuest” software.

### 2.9. Total Cell Lysate Preparation

Following glyphosate (0.1 mM) and TPA (10 nM) treatment to the cells for 24, 48, and 72 h, the medium was aspirated, and the cells were washed twice with cold PBS (10 mM, pH 7.4). Ice-cold lysis buffer (50 mM Tris-HCl, 150 mM NaCl, 1 mM EGTA, 1 mM EDTA, 20 mM NaF, 100 mM Na_3_VO_4_, 0.5% NP-40, 1% Triton X-100, 1 mM PMSF, 10 *μ*g/mL aprotinin, 10 *μ*g/mL leupeptin, and pH 7.4) was added to the plates, which were then placed over ice for 30 min [33]. The cells were scraped, and the lysate was collected in a microfuge tube. The lysates were cleared by centrifugation at 14000 ×g for 15 min at 4°C, and the supernatant (total cell lysate) was either used immediately or stored at −80°C.

### 2.10. Immunoblotting

Immunoblotting was carried in the total cell lysates of HaCaT cells. Proteins (50 *μ*g) were resolved on 10–12% SDS-PAGE, electroblotted onto PVDF membranes, and incubated with antibodies of cyclin D1,cdk 4, cdk 6, cyclin E, cdk 2, IP_3_R, S100A6, S100A9, SOD 1, Bax, Bcl-2, Apaf-1, cytochrome c, caspases 3, 9, and *β*-actin. HRP-conjugated secondary antibodies and chemiluminescence kit (Millipore, USA) were used for detection. Proteins expression was visualized by Versa Doc 4000 MP Imaging System (Bio-Rad Hercules, CA, USA). The intensity of the bands normalized to the band of *β*-actin was measured using software UNSCAN-IT automated digital system version 5.1 (Orem, USA) and then given in terms of calculated quantitative fold change with respect to control.

### 2.11. Statistical Analysis

The data were analyzed to obtain mean values and standard deviation for all treated and vehicle control samples, which were subjected to statistical comparison using student-*t* test; *P* < 0.05 was considered as significant.

## 3. Results

### 3.1. Glyphosate-Induced Proliferation in HaCaT Cells

**Figure 1:**
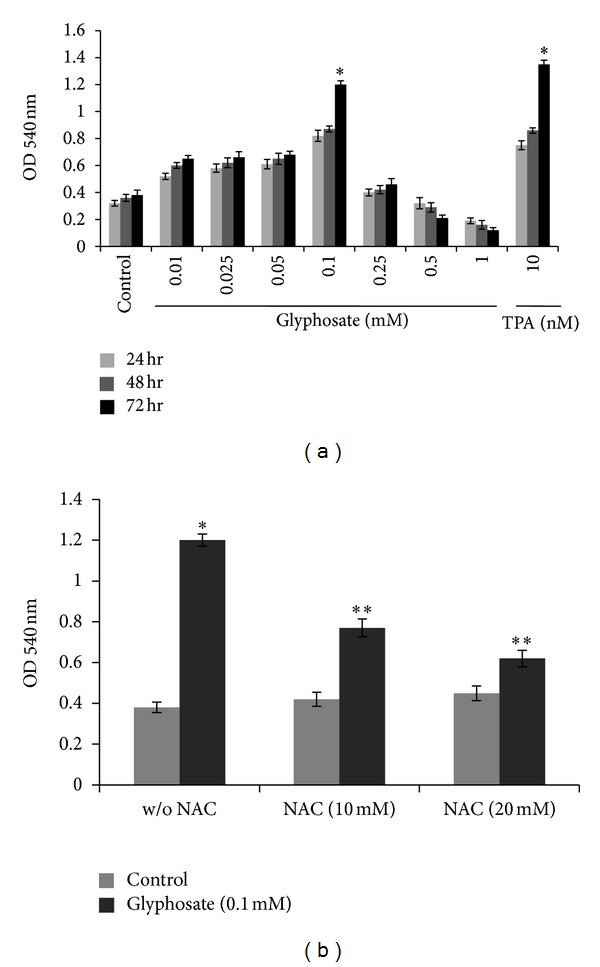
(a) Proliferation effects of glyphosate on HaCaT cells. Cells were exposed to glyphosate (0.01–1 mM) and positive control, and TPA (10 nM) for 24, 48, and 72 h. (b) Inhibition of glyphosate (0.1 mM) induced cell proliferation after 72 h by a prior treatment of NAC (10 mM and 20 mM). OD values from three separate experiments are shown as mean ± SD. *Increase over vehicle control cells (*P* < 0.01); **decrease over glyphosate alone treated cells (*P* < 0.01).

The proliferation rate of HaCaT cells exposed to different concentrations (0.01, 0.025, 0.05, 0.1, 0.25, 0.5, and 1 mM) of glyphosate was evaluated for 24, 48, and 72 h using the MTT assay (Figure 1(a)). Glyphosate at 0.1 mM and lower concentration promoted the growth of HaCaT cells, whereas doses higher than 0.1 mM reduced cell growth in time dependent manner. A significant increase in proliferation was observed in cells exposed to glyphosate at 72 hrs of exposure as compared to the number of viable cells in control at all concentrations except the highest doses (0.25, 0.5, and 1 mM); *P* < 0.01 (Figure 1(a)). 

Proliferation induced by glyphosate in the presence of antioxidant NAC was significantly inhibited compared to glyphosate alone and was not significantly different from control incubated with NAC alone, suggesting that ROS may be involved in glyphosate proliferation effect (Figure 1(b)).

**Figure 2:**
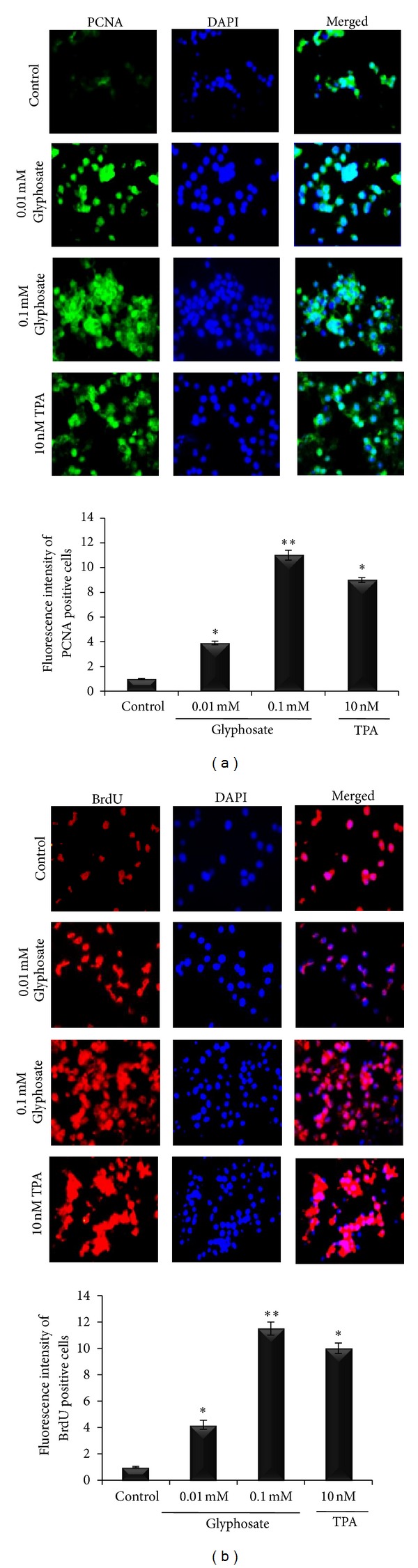
PCNA and BrdU staining of glyphosate and TPA-treated HaCaT cells. HaCaT cells were treated with glyphosate and TPA and were incubated for 72 h. (a) Cells were fixed, and PCNA was detected by immunofluorescence with an anti-PCNA antibody and FITC-labeled secondary antibody (green color). Pictures with green and blue colors were merged. DNA was stained with DAPI (blue color). (b) Cells were pulsed for 4 h with BrdU, fixed, and detected with an anti-BrdU antibody and rhodamine-labeled secondary antibody (red color). DNA was stained with DAPI (blue color). Pictures with red and blue colors were merged. Histogram represents the relative fluorescence intensity of PCNA and BrdU positive cells expressed as a ratio between control and treated HaCaT cells. For all panels, representative data from mean ± SD of at least three independent experiments are shown. *P* < 0.05 versus control cells.

Consistently data from fluorescence microscopy showed increased PCNA expression and a high proportion of BrdU stained cells, indicated a significant (*P* < 0.05) increase in cell proliferation rate in glyphosate (0.1 mM) treated HaCaT cells over control cultures (Figure 2). Thus, proliferative dose of 0.1 mM was used further in all assays.

### 3.2. Glyphosate Decreases Intracellular Ca^2+^[Ca^2+^]_*i*_ Level in HaCaT Cells

**Figure 3:**
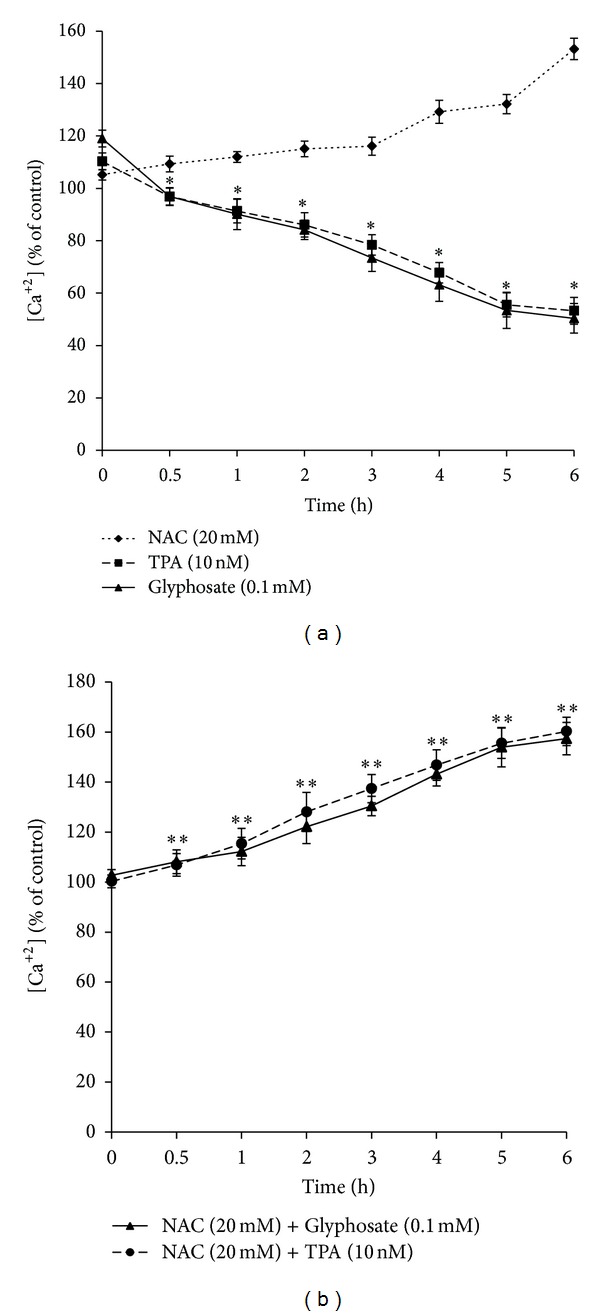
Measurement of glyphosate-induced changes in intracellular Ca^2+^ level in HaCaT cells by spectrofluorometry with Fura-2/AM dye. (a) Ca^2+^ level was measured after treatment with NAC (20 mM), 0.1 mM glyphosate, and 10 nM TPA at 0–6 h. (b) Ca^2+^ level was measured after a prior treatment with 20 mM NAC. Results are mean ± SD from three independent experiments. *Decrease over control cells (*P* < 0.01); **increase over control cells (*P* < 0.01).

To determine whether or not intracellular Ca^2+^ signals promote cell proliferation, we measured the Ca^2+^ concentration in vehicle, positive control, glyphosate, and NAC treated HaCaT cells at regular time intervals (0–6 h). [Ca^2+^]_*i*_ was measured with a calcium indicator dye Fura-2 AM. Incubation of cells with glyphosate (0.1 mM) and TPA (10 nM) resulted in a time-dependent decrease of [Ca^2+^]_*i*_ in HaCaT cells as compared with control cells, *P* < 0.01, whereas NAC increases [Ca^2+^]_*i*_ above control levels (Figure 3(a)). These data suggest that the fall in [Ca^2+^]_*i*_ is due to the decrease Ca^2+^ influx through cell membrane channels. Though, a rapid increase in fluorescence was detected when antioxidant, NAC was added to the cell suspension. From these results, we hypothesize that pretreatment with NAC might cause the opening of Ca^2+^ channels, allowing rapid influx of extracellular Ca^2+^ (Figure 3(b)).

### 3.3. Glyphosate Increases ROS Generation in HaCaT Cells

**Figure 4:**
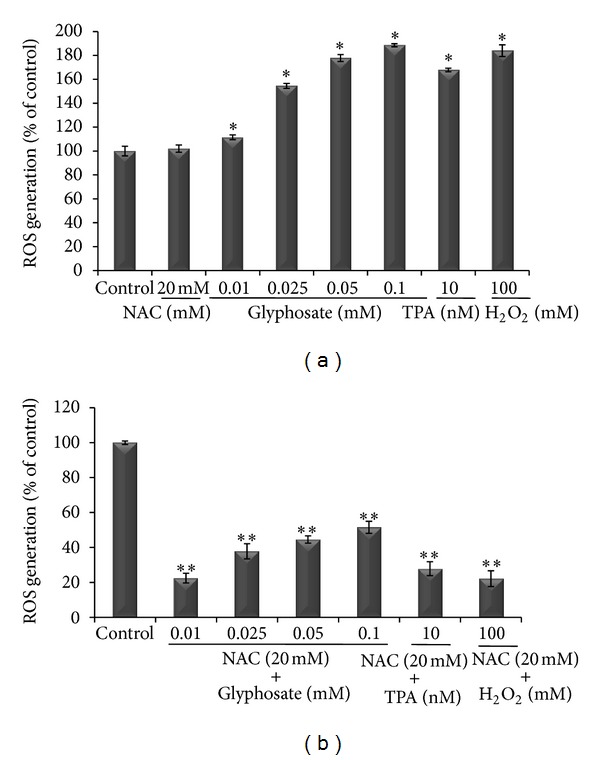
Measurement of glyphosate-induced generation of ROS in HaCaT cells. (a) Generation of ROS was measured using the fluorescent dye (5 (and 6)-chloromethyl-20, 70-dichlorodihydro-fluorescein diacetate acetyl ester) after treatment of HaCaT cells with vehicle alone, NAC (20 mM), (0.01–0.1 mM) glyphosate, and 10 nM TPA. (b) Inhibition of ROS generation after a prior treatment of NAC. Data are expressed as mean ± SD of three independent experiments. *Increase in ROS generation versus control cells (*P* < 0.05); **decrease in ROS generation versus control cells (*P* < 0.05).

Next we sought to test whether glyphosate-elevated [Ca^2+^]_*i*_ is mediated by ROS induction, leading to cell proliferation. To this end, HaCaT cells were exposed to 0-0.1 mM glyphosate, 10 nM TPA, and 100 mM H_2_O_2_ for 24 h. As shown in Figure 4(a), exposure to glyphosate (0.1 mM) increased maximum ROS levels by approximately 90% in HaCaT cells as compared with vehicle control cells. Additionally, ROS generation was similar in glyphosate and positive controls TPA (~70%) and H_2_O_2_ (~80%) treated cells (Figure 4(a)). However, upon pretreatment of the cells with the ROS scavenger NAC in glyphosate (0.1 mM), TPA, and H_2_O_2_ treated cells, ROS generation was abrogated to almost 50%, 70%, and 80% respectively, as compared to that in vehicle control cells, (Figure 4(b)). Moreover, NAC (20 mM) alone treatment has no effect on ROS. These results support that NAC is effective in preventing increase in [Ca^2+^]_*i*_ and oxidative stress resulting from exposure to glyphosate in HaCaT cells. Collectively, these results showed that ROS is involved in the elevation of [Ca^2+^]_*i*_ which in turn is a crucial element in the progression of glyphosate-induced cell proliferation.

### 3.4. Glyphosate-Induced Growth of HaCaT Cells in S-Phase of Cell Cycle and Elevates Expression of G1/S-Phase's Proteins

**Figure 5:**
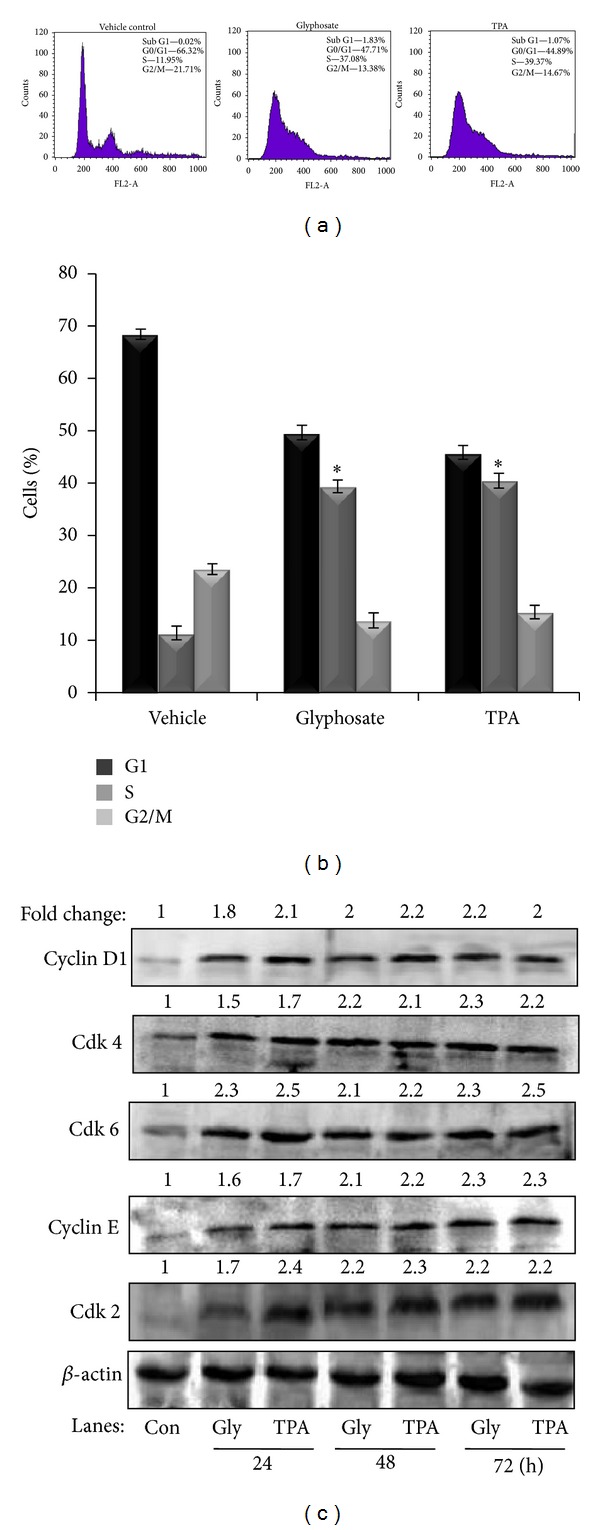
Effect of glyphosate on cell-cycle progression and G1/S-phase proteins in HaCaT cells. Cultured cells were treated with (a) vehicle, glyphosate (0.1 mM), and TPA (10 nM). 24 h later, cell cycle was analyzed by flow cytometry. (b) Quantitative assessment of the percentage of HaCaT cells in G1/S-phases, as indicated by propidium iodide (PI). (c) Immunoblot of G1-S-phase cell-cycle-associated proteins in HaCaT cells after treatment with glyphosate and TPA for 24, 42, and 72 h. All results represent the average of three independent experiments ± S.D. *P < 0.01 compared with the vehicle control.

To determine whether glyphosate-induced cell-cycle progression of HaCaT cells, cell-cycle distribution was analyzed by flow cytometry after exposure to glyphosate for 24 h. Analysis of the cell cycle distributions of HaCaT cells after exposure to glyphosate (0.1 mM) and TPA (10 nM) showed that more cells were in the S-phase of the cell cycle as compared with untreated control cells accompanied by a significant decrease in the G0/G1-phase (Figures 5(a) and 5(b)). To determine if glyphosate-induced growth of HaCaT cells was due to increased activation of the cell-cycle machinery, expression of cell-cycle regulatory proteins was examined using immunoblot assay. Treatment of cells with glyphosate (0.1 mM) and TPA resulted in a time-dependent increase in the expression of cyclin D1, cdk4, cdk6, cyclin E, and cdk2 as compared with vehicle control cells (72 h) (Figure 5(c)). Collectively, these results demonstrate that glyphosate induces expression of G1/S phase's proteins and enhances cell proliferation.

### 3.5. Glyphosate Modulates Ca^2+^ Release Channel, Ca^2+^ Binding, Oxidative Stress, and Apoptosis-Related Proteins

**Figure 6:**
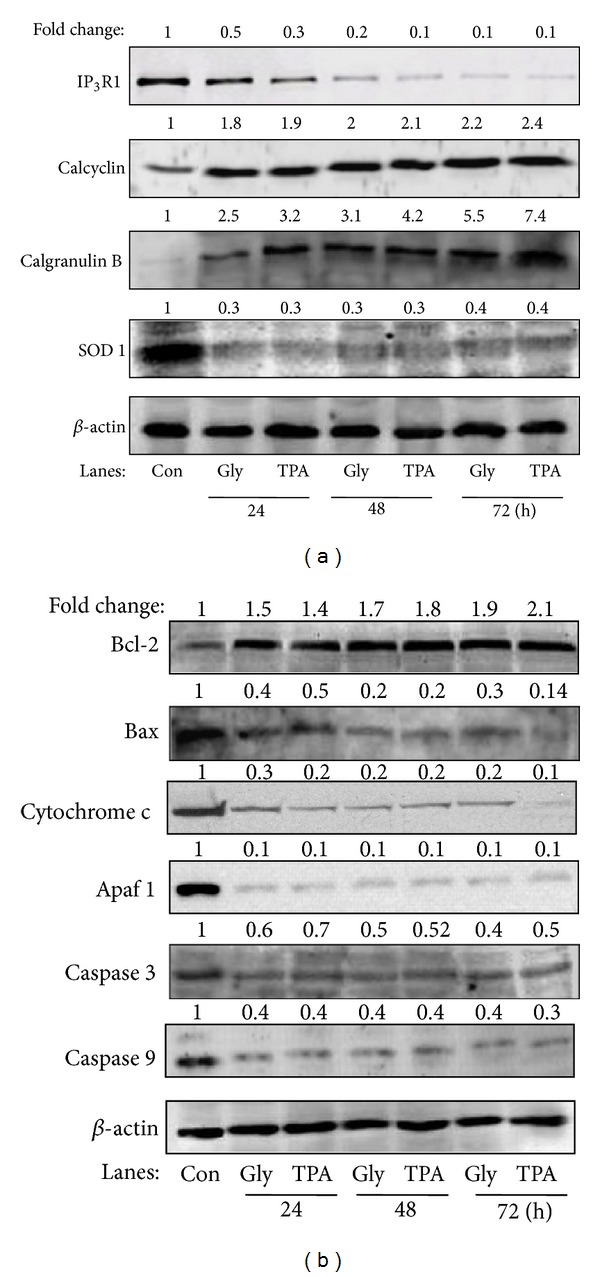
Western blots showing the proliferative effect of glyphosate (0.1 mM) and TPA (10 nM) on the expression levels of (a) Ca^2+^ binding and oxidative stress and (b) apoptosis-related proteins in HaCaT cells for 24, 42, and 72 h. The bands shown here are from a representative experiment repeated three times with similar results. Equal loading was confirmed by stripping the immunoblot and reprobing it for *β*-actin. Quantitative fold change was calculated in respect to control on the basis of pixel density measured by UNSCAN-IT software.

In clarifying the mechanism of glyphosate-induced proliferation in HaCaT cells, we further examined the expression of IP_3_R1 isoform of IP_3_Rs-Ca^2+^ release channel, the proteins that bind Ca^2+^ (S100A6 and S100A9), reduce oxidative stress (SOD 1), and regulate apoptosis (Bax, Bcl-2, cytochrome C, apaf-1, caspases 3, 9) after glyphosate (0.1 mM) and TPA (10 nM) treatment at 24, 48, and 72. In glyphosate treated cells, we found the expression of IP_3_R1 to be hardly detectable at all-time intervals (Figure 6(a)). Little immunoreactivity for S100A6 and S100A9 was visible in vehicle control cultures (72 h) (Figure 6(a)), but dramatic increase of S100A6 and S100A9 expressions was found at designated time points after treatment of glyphosate and TPA (Figure 6(a)). Significant decrease in expression of SOD 1 in glyphosate and TPA exposed cells was also seen in time dependent manner (Figure 6(a)). The immunoreactivity for Bcl-2 was also markedly increased at the same time points posttreatments (Figure 6(b)), whereas Bax, cytochrome C, apaf-1, and caspases 3, 9 expressions were significantly reduced at all-time points after treatment of glyphosate (Figure 6(b)). 

## 4. Discussion

The low reported acute toxicity and short environmental persistence of the herbicide-glyphosate have allowed its worldwide usage in agriculture, even though numerous genotoxic effects have been reported [35–37]. We have previously reported that glyphosate potentially causes tumor promotion in two-stage mouse skin carcinogenesis model, and S100A6, S100A9 (Ca^2+^-regulating proteins), SOD 1 (oxidative stress-related protein) are associated with this tumor promotion [30]. However, the molecular mechanism, how glyphosate contributes in tumor promotion, and regulation of S100A6, S100A9, and SOD 1 remain elusive and require more detailed analysis of the mechanism.

In order to clarify the mode of tumorigenic action of glyphosate, we used human skin keratinocytes HaCaT cells as an *in vitro* model and observed that the imbalance between Ca^2+^ homeostasis and cellular oxidative stress is mainly responsible for glyphosate induced hyperproliferation of HaCaT cells. Using cell proliferation assay, PCNA, and BrdU staining, the effect of glyphosate on cellular proliferation in HaCaT cells was examined. Glyphosate (0.1 mM) significantly increases cell proliferation accompanied with increased PCNA as well as BrdU positivity further suggested its carcinogenic potential (Figures 1 and 2). 

Calcium signalling plays a pivotal role in the regulation of the growth, differentiation, and apoptosis of many kinds of cells including epidermal keratinocytes [38]. Ca^2+^ concentrations may vary with time, even may oscillate (Ca^2+^ influx from intracellular stores and subsequent extracellular Ca^2+^ entry) offering a room for somewhat diverse outcomes such as increased cell proliferation versus differentiation and viability versus death in different types of cells including tumor cells [39]. Considering the importance of Ca^2+^ level in inducing cell proliferation, we measured changes in intracellular Ca^2+^ concentration [Ca^2+^]_*i*_ in glyphosate treated HaCaT cells, respectively, at regular time intervals. Our data demonstrated that control cells maintained [Ca^2+^]_*i*_ while glyphosate significantly decreases [Ca^2+^]_*i*_ in a time dependent manner at proliferation inducing dose for HaCaT cells (Figure 3). Our results are consistent with the findings of Gniadecki and Gajkowska [38] suggesting that emptying intracellular Ca^2+^ stores in keratinocytes (e.g., by a selective blocker of calcium pump, thapsigargin) facilitates basal cell carcinomas or squamous cell carcinomas development. Thus, these results suggest that glyphosate promotes proliferation via modulation of Ca^2+^ levels.

Previously, *in vitro* studies had addressed that glyphosate facilitates oxidative stress [37, 40], and in this study, we confirmed that glyphosate treated HaCaT cells differ from vehicle control cells in the oxidative stress level. The levels of ROS production in glyphosate, TPA, and H_2_O_2_ treated cells were significantly higher than those found in control cells (Figure 4(a)). As oxidative stress and changes in [Ca^2+^]_*i*_ are intimately related, thus, in order to verify the correlation between [Ca^2+^]_*i*_ modifications and the effects of oxidative stress we used NAC, a potent antioxidant which has been demonstrated to be able to promote apoptosis in several cell lines [41, 42]. The antiproliferative effects of 20 mM NAC by reducing ROS have been shown in glioma cells [43]. Here, we observed a substantial decrease in cell proliferation followed by elevation of [Ca^2+^]_*i*_ level and decrease in ROS generation during pretreatment with NAC (20 mM) suggesting ROS has a significant role in the elevation of [Ca^2+^]_*i*_ which in turn is a crucial element in the progression towards cell death as most endonucleases require the presence of Ca^2+^ to cleave DNA strands. These results imply that glyphosate induced cell proliferation by lowering [Ca^2+^]_*i*_ levels was mediated by ROS generation. Therefore, the molecular mechanism for glyphosate induced cell proliferation involves an imbalance in between Ca^2+^ homeostasis and element of oxidative stress, that is, ROS. 

Cell-cycle dysregulation is a hallmark of tumor cells and human cancers. The cell-cycle checkpoints failure causes genetic instability and consequent growth of cancers from the affected cell [44]. Calcium release or decrease in [Ca^2+^]_*i*_ stores might allow the progression from G0 to G1 of cell cycle [45]. Further in this study, we examined the effect of glyphosate on the progression of cell cycle. As shown in Figures 5(a) and 5(b), glyphosate treatment increases cell growth from G1- to S-phase of the cell-cycle as compared with vehicle control cells. Moreover, the results of the present study indicate that glyphosate caused cell cycle acceleration together with an increase in cyclin D1/cdk4, cyclin D1/cdk6, and cyclin E/cdk2, which are involved in cell-cycle progression from G1- to S-phase (Figure 5(c)). Thus, the data clearly showed that glyphosate accumulated cells in S-phase of the cell-cycle concomitant with a decrease in [Ca^2+^]_*i*_ levels and increase in ROS generation. 

Next, we investigated the expression of the proteins that regulate the [Ca^2+^]_*i*_ levels, oxidative stress, and apoptosis. The results of the present study showed a significant increase in two Ca^2+^ binding proteins, S100A6, and S100A9 (Figure 6(a)). In the past, we studied alterations in global protein expression using samples of mouse skin treated with glyphosate and the tumor promoter TPA, which is a potent activator of keratinocyte proliferation [46], epidermal hyperplasia and dermal inflammation [47], and we found strong upregulation of S100A6 and S100A9 [30]. More recently, strong upregulation of both proteins was also reported by us in HaCaT cells upon mancozeb (fungicide) exposure [48]. Numerous recent reviews focused on the extracellular function of S100A6/A9 and its presumed impact on pathological processes such as acute and chronic inflammations or carcinogenesis [49–51]. Ca^2+^ binding prompts a conformational variation in the S100A6 molecule which in result raises its total hydrophobicity and lets the target proteins and transduction of Ca^2+^ signals collaborate [52]. However, the crosstalk between Ca^2+^ level changes and these Ca^2+^ binding proteins in cancer progression remains elusive. Glyphosate treatment also significantly reduced expression of antioxidant SOD 1 protein in HaCaT cells thus can be associated with stimulation of oxidative stress (Figure 6(a)). These results suggest that glyphosate treatment could cause intracellular Ca^2+^ imbalance, induce cellular oxidative stress, and modify S100A6/A9 and Cu/Zn-SOD 1 expressions, a phenomenon that may contribute to the mechanisms involved in HaCaT cell proliferation.

**Figure 7:**
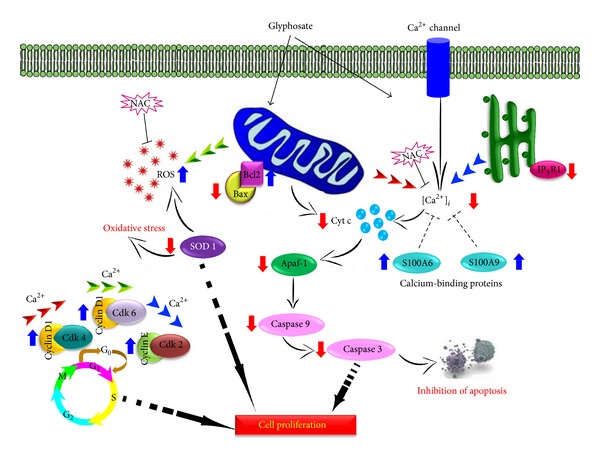
Proposed itinerary for glyphosate-induced proliferation in human skin keratinocytes HaCaT cells.

In a cellular system, one of the two foremost ways of Ca^2+^ discharge from the Ca^2+^ store is the IP_3_Rs, the other being ryanodine receptor (RyR). Essential to an understanding of Ca^2+^ signaling hence is gratitude of the control of IP_3_Rs-mediated Ca^2+^ release [53, 54]. Three mammalian isoforms of IP_3_R exist, namely, IP_3_R1, IP_3_R2, and IP_3_R3 known to play a key role in promoting different signaling prospects during changes in Ca^2+^ levels. One intriguing factor is the proximity of ER to mitochondria that may facilitate the mitochondrial overload of Ca^2+^ released from the IP_3_Rs with certain apoptotic stimuli, triggering the opening of the mitochondrial permeability transition pore and the release of apoptotic signaling molecules, such as cytochrome c and apoptosis-inducing factor, which leads to the activation of caspases [55, 56]. Moreover, several key components of apoptotic cascades, such as cytochrome c [57] and anti-apoptosis proteins Bcl-2 [58, 59] and Bcl-X_L_ [60], have been reported to interact with the internal coupling domain and/or the COOH-terminal tail of IP_3_R and enhance the Ca^2+^ release activity of IP_3_Rs during apoptosis. It has been shown that less apoptosis occurs in cells in which IP_3_R1 expression was reduced or wholly silenced [61]. Similarly, in our case the decrease in the level of IP_3_R1 also observed upon glyphosate exposure in HaCaT cells thus prevented cell death [62]. Furthermore, in the cancer cells the amplified appearance of antiapoptotic members of the Bcl-2 family of proteins or reduced appearance of the proapoptotic proteins like Bax or Bak can shield these cells from apoptosis by controlling [Ca^2+^]_*i*_ signals [63, 64]. This mechanism was also observed in our case as we found a significant increase in Bcl-2 and decrease in Bax proteins simultaneously with a decrease in [Ca^2+^]_*i*_ levels (Figure 6). The rise of [Ca^2+^]_*i*_ has been demonstrated to cause mitochondrial inner transmembrane potential collapse and release of cytochrome c, activation of caspase 3, and then apoptosis [41]. In contrast, in the present study, we found that lowering [Ca^2+^]_*i*_ with glyphosate treatment suppressed the activation of caspases and thereby possibley preventing apoptosis. The results of our studies can be summarized in a schematic presentation (Figure 7).

## 5. Conclusion

In conclusion, in this study, we demonstrated that glyphosate may possibly exert proliferative effect in HaCaT cells by activating Ca^2+^ binding proteins to promote the imbalance of intracellular Ca^2+^ homeostasis and lessen SOD1 to increase ROS generation. This effect was partially reversed by treatment with antioxidant NAC indicating connections between oxidative stress and hypocalcaemia. Reduced Ca^2+^ levels enhance Bcl-2 and decrease Bax, subsequently leading to decrease in cytochrome c to stimulate further decrease of caspase 3 via the downregulation of IP_3_R1 level, thus halting apoptosis. The present study for the first time provides insight into the mechanism of glyphosate-induced neoplastic potential in mammalian skin system.
